# Assessment of glycemic variability and lifestyle behaviors in healthy nondiabetic individuals according to the categories of body mass index

**DOI:** 10.1371/journal.pone.0291923

**Published:** 2023-10-04

**Authors:** Kazuhiro Kashiwagi, Jun Inaishi, Shotaro Kinoshita, Yasuyo Wada, Sayaka Hanashiro, Kiko Shiga, Momoko Kitazawa, Shiori Tsutsumi, Hiroyuki Yamakawa, Junichiro Irie, Taishiro Kishimoto

**Affiliations:** 1 Hills Joint Research Laboratory for Future Preventive Medicine and Wellness, Keio University School of Medicine, Tokyo, Japan; 2 Center for Preventive Medicine, Keio University School of Medicine, Tokyo, Japan; 3 Division of Endocrinology, Metabolism and Nephrology, Department of Internal Medicine, Keio University School of Medicine, Tokyo, Japan; 4 Graduate School of Interdisciplinary Information Studies, The University of Tokyo, Tokyo, Japan; 5 Department of Health Promotion, National Institute of Public Health, Saitama, Japan; 6 Department of Neuropsychiatry, Keio University School of Medicine, Tokyo, Japan; 7 Department of Clinical Psychology, Faculty of Human Relations, Shigakukan University, Kagoshima, Japan; 8 Graduate School of Health Management, Keio University, Kanagawa, Japan; 9 Department of Psychiatry, The Zucker Hillside Hospital, Northwell Health, New York, NY, United States of America; 10 Department of Psychiatry and Department of Molecular Medicine, Donald and Barbara Zucker School of Medicine at Hofstra/Northwell, New York, NY, United States of America; Hamasaki Clinic, JAPAN

## Abstract

**Background:**

There are limited data about the association between body mass index (BMI), glycemic variability (GV), and life-related factors in healthy nondiabetic adults.

**Methods:**

This cross-sectional study was carried out within our ethics committee-approved study called “Exploring the impact of nutrition advice on blood sugar and psychological status using continuous glucose monitoring (CGM) and wearable devices”. Prediabetes was defined by the HbA1c level of 5.7–6.4% and /or fasting glucose level of 100–125 mg/dL. Glucose levels and daily steps were measured for 40 participants using Free Style Libre and Fitbit Inspire 2 under normal conditions for 14 days. Dietary intakes and eating behaviors were assessed using a brief-type self-administered dietary history questionnaire and a modified questionnaire from the Obesity Guidelines.

**Results:**

All indices of GV were higher in the prediabetes group than in the healthy group, but a significant difference was observed only in mean amplitude of glycemic excursions (MAGE). In the multivariate analysis, only the presence of prediabetes showed a significant association with the risk of higher than median MAGE (Odds, 6.786; 95% CI, 1.596–28.858; P = 0.010). Additionally, the underweight (BMI < 18.5) group had significantly higher value in standard deviation (23.7 ± 3.5 vs 19.8 ± 3.7 mg/dL, P = 0.038) and coefficient variability (22.6 ± 4.6 vs 18.4 ± 3.2%, P = 0.015), compared to the normal group. This GV can be partially attributed to irregularity of eating habits. On the contrary, the overweight (BMI ≥ 25) group had the longest time above the 140 or 180 mg/dL range, which may be due to eating style and taking fewer steps (6394 ± 2337 vs 9749 ± 2408 steps, P = 0.013).

**Conclusions:**

Concurrent CGM with diet and activity monitoring could reduce postprandial hyperglycemia through assessment of diet and daily activity, especially in non- normal weight individuals.

## Introduction

Obesity (body mass index (BMI) ≥25) has been consistently reported to be associated with the high risk of type2 diabetes mellitus (T2DM) [1, 2]. On the contrary, a large Japanese cohort study showed that underweight in adults aged 60–79 years may be associated with the risk of T2DM [3]. Jung et al reported that underweight, overweight (≥ 23 and ≺ 25), obese (≥ 25 and < 30), and severe obese (≥ 30) group had the higher hazard ratios for T2DM than normal group during follow up for 10 years [4]. Among Japanese women without parental DM history, combining “low” (< 25th percentile) BMI at age 18 years with current “middle” (25th to 74th percentile) or “high” (> 75th percentile) BMI had significantly high odds ratios (2.25 or 13.92) for adult-onset DM [5]. Furthermore, low BMI was associated with adverse coronary heart disease outcomes in Asian populations [6].

Continuous glucose monitoring (CGM), which can collect glucose data for several days in a non-invasive way, has been developed for diabetics to estimate and control their plasma glucose changes throughout the day. Prior studies have evaluated sensor glucose levels and glycemic variability (GV) in healthy individuals without diabetes [7–9]. Many self-reported non-diabetic participants have frequent glycemic excursions into the diabetic range: Fifteen % of healthy and 36% of prediabetic individuals had glucose levels above 200 mg/dl on CGM [10]. The quantity of carbohydrate has been shown to be a consistent predictor of postprandial blood glucose levels [11]. Moreover, not only type and amount of meals but also eating behaviors including eating frequency, skipping breakfast, snacking and eating speed, influence the onset of diabetes [12–14]. In a recent review, there are several reports on the association between sedentary time or exercise and glycemic excursions in patients with type 2 diabetes [15]. However, there are limited data combining CGM-measured glucose levels with diet and physical activity that could impact glycemia in individuals without diabetes [16].

We hypothesized that non-normal weight (underweight or overweight/obesity) people may be less active, and their eating behaviors such as skipping breakfast or eating fast may lead to greater GV, compared to normal weight people. The aim of this study is to examine the association between BMI, GV, and life-related factors such as diet, eating behaviors and daily activity in healthy non-diabetic individuals under normal conditions.

## Materials and methods

### Trial design

The present study was carried out within our ethics committee-approved study called “Exploring the impact of nutrition advice on blood sugar and psychological status using CGM and wearable devices: A feasibility study”. Briefly, this original research exploratory examined the relationship between blood glucose variability, activities of daily living obtained from wearable devices and psychological state obtained from questionnaires and ecological momentary assessment. Also, the extent to which intervention effects of advice on general diet and eating behavior influences these outcomes has been verified. Therefore, participants were monitored by CGM for two weeks before and after the dietary advice by our registered dietitian. However, in this cross-sectional study, we aimed to examine the association between BMI, GV, and life-related factors under normal conditions. In other words, this study is from the original clinical trial and the results presented from this cross-sectional trial are from the baseline prior to deploying the study intervention. The original research was approved by the institutional Review Board of Keio University Hospital (IRB No. 20211103) and has been registered in University Hospital Medical Information Network (UMIN) Clinical Trial Registry (UMIN000046858). Study participants signed a consent form for the study.

### Participants

Healthy office workers without diabetes (previous diagnosis of diabetes or hemoglobin A1c (HbA1c) ≥ 6.5% or fasting plasma glucose ≥ 126 mg/dL), who had undergone a medical health check-up within 1 year were recruited via internal company communication in February 1, 2022 to February 28, 2022. A total of 40 individuals with a 1:1 male to female ratio were recruited for this research. All participants provided written informed consent before enrolling in the research. Prediabetes was defined by the HbA1c level of 5.7 to 6.4% and /or impaired fasting glucose level of 100 to 125 mg/dL [17] by the medical health check-up record. BMI was calculated as weight (kg) divided by the square of the body height (m). They were instructed not to change their usual diet and physical activity until receiving general dietary advice. Thus, the present study period was defined as the first 14 days during CGM monitoring. Data collection occurred between February and April 2022.

### CGM procedures and measurement of glycemic variability

Glucose levels were assessed by using Freestyle Libre (Abbot, Tokyo, Japan), intermittent**-**scanning CGM. The sensor measures the glucose concentration in the interstitial fluid every 15 minutes for a period of 14 days. Sensor data was downloaded, processed, visualized and archived using a licensed software. Only those who covered more than 60% of CGM monitoring over 14 days were included in the final analysis.

As the currently adopted in the consensus on the use of CGM metrics [18, 19], the mean glucose level, standard deviation (SD), percent coefficient variability (CV), mean amplitude of glycemic excursions (MAGE), time above range (TAR), time in range (TIR), or time below range were calculated by means of software (Excel, Microsoft Office). We evaluated the GV as SD, CV and MAGE, and the definitions and interpretation of each index were described in previous report [20].

### Evaluation of dietary history and behavior

For an assessment for daily food and nutrient intakes, a brief-type self-administered diet history questionnaire (BDHQ) consisting of 58-item food frequency questionnaires and 15-item diet history questionnaires [21, 22] was used. In addition, for the assessment for eating behavior, questionnaires from the Guideline for Obesity issued by the Japan Society for the Study of Obesity [23, 24] were excerpted and partially modified. It comprises 36-item detailed questions contained in the following 7 major categories: G1) recognition for weight and constitution, G2) external eating behavior, G3) emotional eating behavior, G4) sense of hunger, G5) eating style, G6) food preference, G7) regularity of eating habits. All items were rated on a scale of 1 (I don’t think so at all), 2 (I don’t think so), 3 (I think so a little), and 4 (I think), and the average score was calculated for each item. We used responses to three, two or seven questions in the G5, G6 or G7 categories, respectively, that were thought to affect blood glucose levels. Both questionnaires were examined at the end of the study.

### Assessment of activity

Participants were instructed to wear a smartwatch-type activity tracer, Fitbit Inspire 2 (Fitbit Inc., Tokyo, Japan) [25], throughout the day, except when bathing. This activity monitor communicated with a smartphone application to provide feedback on the number of steps taken. The average step counts per day were calculated for each participant. For the final analysis, we used data of participants who covered at least 60% of their Fitbit wearing time, excluding sleep time, for 14 days.

### Statistical analysis

All participants were included in the CGM analysis, but only one with normal weight was excluded from the analysis of daily activity due to lack of data by activity tracer. For continuous data, mean values were expressed with SD, and statistical differences between two groups as the reference of the normal weight group, were determined using the *t*-test or Mann-Whitney U., when the data was normally distributed or not, respectively. For categorical data, numbers were presented with percentage, and statistical differences were determined using the chi-square tests. To rule out multicollinearity, we ensured the absolute value of the correlation coefficient between the independent variables before regression analysis. Then, factors associated with the risk of higher than median MAGE (MAGE ≥52) by CGM were analyzed using logistic regression analysis. For sensitivity analyses, similar comparison of CGM metrics between two groups were performed by using the cutoff point of BMI of 23 kg/m^2^ in Asian-Pacific obesity guideline defined by the World Health Organization International Obesity Task Force for Asians (underweight ≺ 18.5, 18.5 ≤ normal weight ≺ 23, and overweight ≥ 23 kg/m^2^ [26]. All statistical analyses were performed using SPSS software version 24 (SPSS, Inc., Chicago, Ill). All *p*-values less than 0.05 were considered statistically significant.

## Results

### CGM metrics (GV and percentage of glucose sensor values) in healthy or prediabetes participants

Of the 40 healthy nondiabetic participants, 20 were female and 14 were prediabetic, with a mean age of 40.3 years. The average valid time of the Libre and Fitbit sensors was good at 92.2% and 88.0%, respectively, of the entire study period. In this study, all participants were included in the final analysis. Overall GV values and percentage of glucose sensor values are shown in **Table 1**. The prediabetes group was older than the healthy group, but there was no gender difference. All indices of GV were higher in the former group than in the latter group, but a significant difference was observed only in MAGE. Percentage of TAR (180) and TAR (140) were higher in the prediabetes group, compared to the healthy group, but there was no significant difference. Interestingly, 13 healthy participants (50%) had TAR (180) > 0, compared with 71% with prediabetes. We next investigated factors related to the risk of higher than median MAGE (MAGE ≥52) by CGM. In both Model 1, which included age, sex and the presence of prediabetes, and Model 2, which further adjusted for BMI, only the presence of prediabetes showed significant difference (Odds, 6.786; 95% CI, 1.596–28.858; P = 0.010) (**Table 2**).

**Table 1:** Summary of CGM metrics by presence of prediabetes in healthy non-diabetes participants (n = 40).

Characteristics of research participants	Healthy (n = 26)	Prediabetes (n = 14)	P
Male (%)	12 (46%)	8 (57%)	0.507
Age, year (range)	33.9 ± 9.2 (23∼56)	52.3 ± 5.2 (45∼60)	**0.000**
BMI, kg/m^2^ (range)	21.1 ± 2.8 (16.9∼28)	21.5 ± 2.1 (18.5∼25.1)	0.660
FBS, mg/dL (range)	84.2 ± 6.9 (67∼97)	101.9 ± 9.2 (84∼117)	**0.000**
HbA1c, % (range)	5.0 ± 0.2 (4.4∼5.5)	5.6 ± 0.3 (5.0∼6.1)	**0.000**
**Overall glucose distribution and variability**			
Mean, mg/dL	104.1 ± 20.7	110.0 ± 8.7	0.316
SD, mg/dL	20.0 ± 4.1	22.1 ± 4.4	0.151
CV, %	18.2 ± 3.9	20.1 ± 3.7	0.163
MAGE, mg/dL	49.2 ± 9.4	56.0 ± 11.4	**0.044**
**Percentage of glucose sensor values**			
TAR (180), %	0.9 ± 1.3	1.6 ± 1.5	0.086
TAR (180) ≻ 0, %	13 (50%)	10 (71%)	0.191
TIR (70–180), %	97.6 ± 2.8	97.8 ± 1.3	0.832
TAR (140), %	8.0 ± 7.1	10.6 ± 6.2	0.253
TIR (70–140), %	90.5 ± 7.2	88.6 ± 5.9	0.407
**Total energy, nutrients, food groups**			
Energy, kcal/d	1482 ± 380	1814 ± 587	**0.037**
Carbohydrate, % energy	48.3 ± 6.9	46.9 ± 7.7	0.556
Alcohol, % energy	6.4 ± 7.0	5.0 ± 8.0	0.570
Confections, g/d	59.2 ± 45.7	62.5 ± 40.7	0.823
Sugar sweetened beverages, g/d	55.7 ± 73.8	97.1 ± 101.7	0.193
**Lifestyle behaviors**			
G5: Do you eat fast?	2.9 ± 0.8	2.8 ± 01.0	0.645
G6: Do you often eat snacks?	2.2 ± 0.9	1.9 ± 0.9	0.313
G6: Do you often drink?	2.2 ± 0.9	1.9 ± 0.9	0.313
G7: Do you often skip breakfast?	2.1 ± 1.1	1.6 ± 1.2	0.237
G7: Do you eat between meals during the day?	2.6 ± 0.9	1.9 ± 0.8	**0.023**
G7: Do you eat a late-night snack?	2.0 ± 0.9	1.3 ± 0.5	**0.011**
**Dairy activity (n = 25, 14)**			
Average dairy step counts	9172 ± 2056	9446 ± 3495	0.797

CGM, continuous glucose monitoring; SD, standard deviation; CV, coefficient variation; MAGE, mean amplitude of glycemic excursions; TAR, time above range; TIR, time in range. Prediabetes was defined by the HbA1c level of 5.7 to 6.4% and /or impaired fasting glucose level of 100 to 125 mg/dL.

**Table 2:** Multiple logistic regression analysis for the association between characteristics of the participants or lifestyle behaviors and the risk of higher than median MAGE (MAGE ≥52) by CGM.

		Odds	95% CI	P
**Model1**	sex			0.501
	age			0.293
	Prediabetes	6.786	1.596–28.858	**0.010**
**Model2**	sex			0.501
	age			0.293
	Prediabetes	6.786	1.596–28.858	**0.010**
	BMI			0.874

MAGE, mean, amplitude of glycemic excursions; CGM, continuous glucose monitoring; Prediabetes was defined by the HbA1c level of 5.7 to 6.4% and /or impaired fasting glucose level of 100 to 125 mg/dL.

### CGM metrics by BMI classification

**Fig 1:**
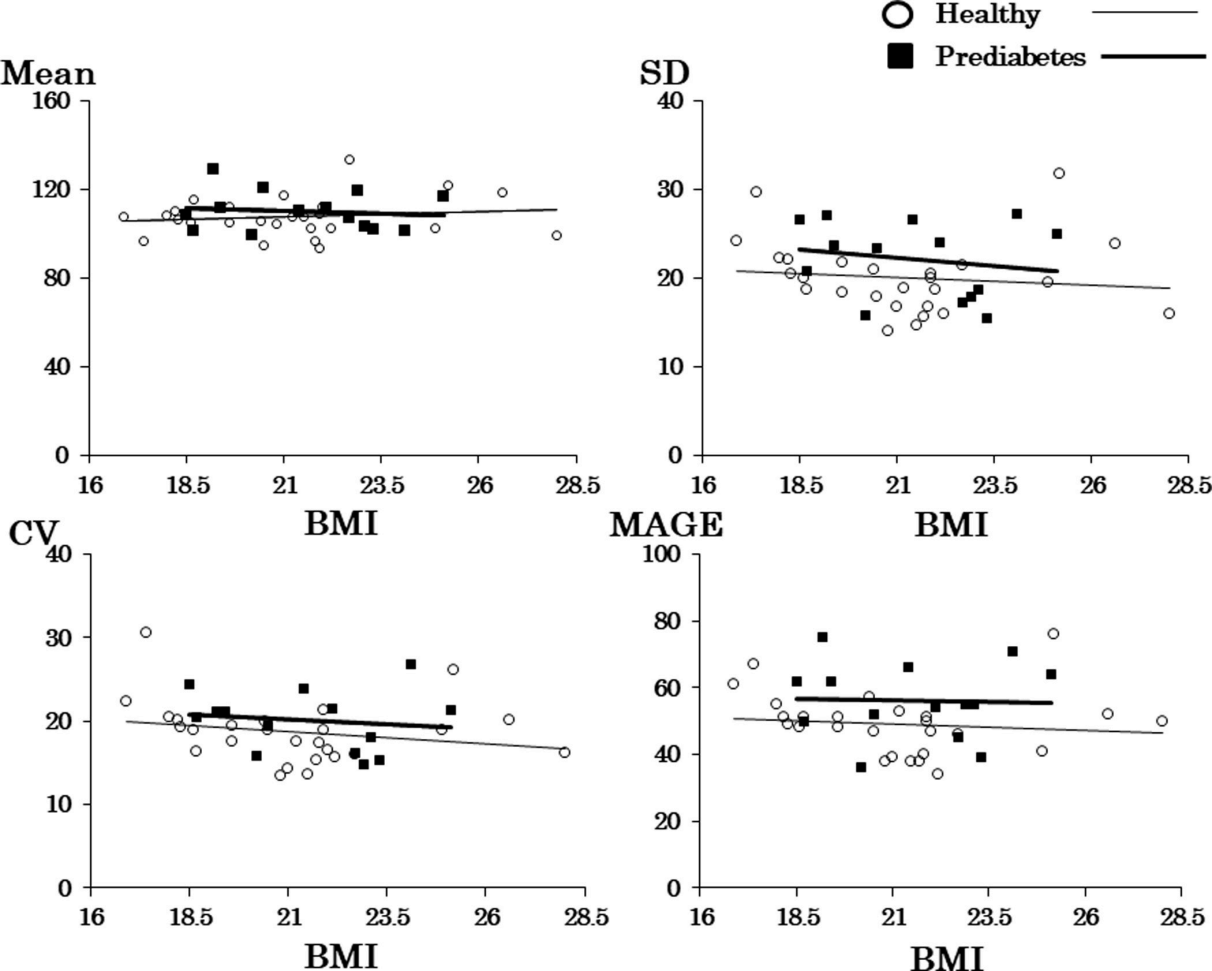
Scatter plots showing CGM metrics and BMI in the prediabetes group and the healthy group. SD, standard deviation; CV, coefficient variation; MAGE, mean amplitude of glycemic excursions.

**Fig 1** displays scatter plots for mean glucose concentration and each of the three GV indices, with closed squares and bold regression lines corresponding to prediabetes and open circles and thin regression lines to healthy participants. The bold lines for the prediabetes groups are always above the thin lines for the healthy group, except the upper left figure. Except for the upper left scatter plot of BMI and mean glucose concentration, the values for each GV index seem to be lowest around BMI of 21 kg/m^2^.

All 40 participants were grouped into underweight (n = 5), normal weight (n = 31) and overweight (n = 4) (**Table 3**). Compared to the normal weight group, the underweight group consisted only of women, and 13 and 1 prediabetic participants were included in the standard and overweight group, respectively. The underweight group had the lowest mean glucose concentration among the three groups, but had higher indices of GV (SD, CV and MAGE) than the normal weight group, especially significantly higher SD and CV. As for glucose sensor values, percentage of TAR (180) and TAR (140) were highest in the overweight group. Interestingly, all participants in the underweight group had TAR (180) > 0, compared to 48% in the normal weight group (P = 0.041). Compared to the normal weight group by the cutoff point of BMI of 23 kg/m^2^, the underweight group also had significantly higher SD and CV, and percentage of TAR (180) and TAR (140) were also highest in the overweight group (**S1 Table**).

**Table 3:** Summary of CGM metrics by BMI classification in healthy non-diabetes participants.

BMI	Underweight	Normal weight	Overweight	P		
	(< 18.5)	(18.5 ≦ < 25)	(≥ 25)			
**Characteristics of participants**	Gr1 (n = 5)	Gr2 (n = 31)	Gr3 (n = 4)	Gr1 vs Gr2	Gr1 vs Gr3	Gr2 vs Gr3
Male, %	0 (0%)	18 (58%)	2 (50%)	**0.016**	0.167	0.581
Age, year	35.4 ± 11.3	40.5 ± 12.4	45.3 ± 8.6	0.399	0.194	0.461
BMI, kg/m^2^	17.8 ± 0.6	20.8 ±1.4	25.0 ± 1.6	**0.000**	**0.000**	**0.000**
FBS, mg/dL	86.6 ± 6.8	91.0 ± 12.5	89.8 ± 8.3	0.844	0.550	0.844
HbA1c, %	5.1 ± 0.3	5.3 ± 0.4	5.4 ± 0.3	0.297	0.110	0.441
Prediabetes, (%)	0 (0%)	13 (42%)	1 (25%)	0.089	0.444	0.470
**Overall glucose distribution and variability**						
Mean, mg/dL	105.8 ± 5.2	108.1 ± 9.0	114.0 ± 10.3	0.599	0.163	0.228
SD, mg/dL	23.7 ± 3.5	19.8 ± 3.7	24.2 ± 6.5	**0.038**	0.897	0.054
CV, %	22.6 ± 4.6	18.4 ± 3.2	21.0 ± 4.1	**0.015**	0.598	0.153
MAGE, mg/dL	56.6 ± 7.4	49.7 ± 10.2	60.5 ±12.0	0.154	0.567	0.057
**Percentage of glucose sensor values**						
TAR (180), %	1.2 ± 0.4	0.9 ± 1.3	2.5 ± 2.5	0.649	0.287	**0.046**
TAR (180) > 0, %	5 (100%)	15 (48%)	3 (75%)	**0.041**	0.444	0.323
TIR (70–180), %	95.0 ± 4.6	98.2 ± 1.5	97.3 ± 3.0	0.203	0.431	0.309
TAR (140), %	8.2 ± 1.1	8.2 ± 7.0	14.8 ± 7.9	0.994	0.197	0.092
TIR (70–140), %	87.6 ± 5.2	90.8 ± 6.7	85.3 ± 7.9	0.314	0.607	0.134
**Life style related factors**						
**Energy** (kcal/d)	1406 ±180	1606 ± 409	1834 ±721	0.358	0.353	0.190
< Estimated energy requirement (PAL I)	4 (80%)	28 (90%)	3 (75%)	0.466	0.556	0.687
**Nutrients** (% energy)						
Protein (% energy)	14.8 ± 0.5	15.7 ± 2.3	17.8 ± 3.5	0.447	0.086	0.087
Fat (% energy)	28.3 ±1.8	29.4 ± 5.2	29.2 ± 2.0	0.358	0.505	0.915
Carbohydrates (% energy)	51.8 ± 4.2	47.2 ± 7.3	47.0 ± 8.6	0.183	0.305	0.955
Alcohol (% energy)	4.0 ± 5.1	6.4 ± 7.6	4.2 ± 8.4	0.496	0.970	0.583
**Food groups** (g/d)						
Rice (g/d)	129.9 ± 64.1	216.3 ± 118.3	218.9 ± 125.2	**0.036**	0.257	0.969
Bread (g/d)	36.9 ± 21.2	32.2 ± 23.8	33.1 ± 32.3	0.679	0.838	0.942
Noodles (g/d)	93.1 ± 41.6	55.8 ± 37.8	86.1 ± 68.2	0.051	0.853	0.179
Confections (g/d)	73.4 ± 36.4	61.1 ± 46.2	38.0 ± 24.1	0.576	0.140	0.336
Meat (g/d)	64.7 ± 2.4	84.4 ± 36.9	82.0 ± 12.3	**0.006**	0.064	0.901
Fish and shellfish (g/d)	41.9 ± 15.1	63.5 ± 27.9	88.8 ± 48.3	0.103	0.146	0.127
Vegetables (g/d)	205.2 ± 39.3	212.3 ± 106.9	171.4 ± 37.3	0.885	0.231	0.457
Fruits (g/d)	85.2 ± 36.2	91.8 ± 90.0	90.5 ± 65.4	0.873	0.881	0.977
Milk and milk products (g/d)	87.6 ± 102.9	102.2 ± 75.6	122.3 ± 50.8	0.705	0.560	0.611
Sugar sweetened beverages (g/d)	13.3 ± 13.3	74.7 ± 85.7	106.5 ± 120.2	**0.001**	0.219	0.508
**Eating Behaviors G5: eating style**						
Do you eat fast?	2.4 ± 0.5	2.9 ± 0.9	3.3 ± 1.0	0,240	0.136	0.479
Do you eat without chewing too much?	2.2 ± 0.4	2.9 ± 0.8	3.0 ± 0.8	0.066	0.101	0.754
Do you have a lot of mouthfuls?	2.0 ± 0.7	2.7 ± 0.8	3.0 ± 0.8	0.081	0.089	0.450
**Eating Behaviors G6**: **food preference**						
Do you often eat snacks?	2.0 ± 0.7	2.0 ± 0.9	2.5 ± 1.3	1.000	0.480	0.305
Do you often drink?	2.0 ± 0.7	2.0 ± 0.9	2.5 ± 1.3	1.000	0.480	0.306
**Eating Behaviors G7: regularity of eating habits**						
Do you often skip meals during the day?	2.6 ± 1.5	2.0 ± 1.0	2.0 ± 0.8	0.286	0.502	0.952
Do you often skip breakfast?	2.4 ± 1.5	1.9 ± 1.0	1.5 ± 1.0	0.360	0.343	0.471
Do you have irregular mealtimes?	2.8 ± 0.8	2.6 ± 0.8	2.5 ± 0.6	0.617	0.563	0.777
Don’t you have time to eat slowly?	3.2 ± 0.8	2.5 ± 0.7	2.5 ± 1.0	**0.032**	0.289	0.899
Do you eat between meals during the day?	3.2 ± 0.8	2.3 ± 0.9	1.8 ± 1.0	**0.043**	**0.046**	0.229
Do you eat a late-night snack?	2.2 ± 0.8	1.7 ± 0.9	1.3 ± 0.5	0.291	0.087	0.292
Do you often drink canned juice, canned coffee, or energy drinks?	1.6 ± 0.5	1.8 ± 0.9	1.5 ± 1.0	0.554	0.853	0.471
**Dairy Activity (n = 5, 30, 4)**						
Average dairy step counts	8648 ± 2572	9749 ± 2408	6394 ± 2337	0.355	0.217	**0.013**
Average daily step counts ≥ 10,000	2 (40%)	14 (47%)	0 (0%)	0.585	0.278	0.104

CGM, continuous glucose monitoring; SD, standard deviation; CV, coefficient variation; MAGE, mean amplitude of glycemic excursions; TAR, time above range; TIR, time in range. Prediabetes was defined by the HbA1c level of 5.7 to 6.4% and /or impaired fasting glucose level of 100 to 125 mg/dL.

### Comparison of diet history by BMI classification (Table 3)

Total energy intakes increased in the higher BMI group, but the ratio of each of the three macronutrients to total energy intake was approximately the same. Compared to the normal weight group as a reference, the overweight group had higher intake of some kind of foods such as sugar sweetened beverages, but they did not differ between the two groups. On the other hand, the underweight group showed lower intake of most kind of foods, except noodles and confections, than the reference group.

### Comparison of eating behavior and daily activity by BMI classification (Table 3)

Each score in the G5 and G6 category increased in the higher BMI group. Compared to the normal weight group, the underweight group scored higher on most questions for the G7 category, especially lack of time to eat slowly and eating between meals during the day (3.2 ± 0.8 vs 2.5 ± 0.7, P = 0.032; 3.2 ± 0.8 vs 2.3 ± 0.9, P = 0.043). On the contrary, there was a significant difference in average daily step counts between the overweight group and the reference (6394 ± 2337 vs 9749 ± 2408 steps, P = 0.013).

## Discussion

This is, to the best of our knowledge, the first study to investigate the association between BMI, GV, and life-related factors including diet, eating behaviors and daily activity, in healthy non-diabetic individuals under normal real-life conditions. Here, we demonstrated the impact of BMI on GV and the possibility of relationship between lifestyle factors and GV. Our results highlight that underweight (BMI < 18.5 kg/m^2^) group had the significantly higher value in SD and CV in comparison with normal weight group (18.5 ≤ BMI ≺ 25 kg/m^2^) as a reference, although the underweight group had the lowest mean value among the three groups. Results from BDHQ and the eating behavior questionnaire suggest that this GV could be partially caused by irregularity of eating habits including habitual eating between meals during the day. On the other hand, overweight group had highest percentage of TAR (180) and TAR (140) among the three groups, which may be due to eating style (i.e., eating fast) and taking fewer steps.

Insulin resistance and impaired insulin secretion are the two main components in the pathophysiology of T2DM. The predominant mechanism in lean diabetic patients was impaired insulin secretion, whereas that for obese subjects was insulin resistance [27]. Hyperglycemia is a causative factor for β-cell dysfunction before the onset of diabetes [28], and increased GV was related with decreased oral disposition index, a useful marker of islet β-cell function [29]. Thus, using CGM to assess the extent of postprandial hyperglycemia and GV in healthy individuals without diabetes is of significance for prediction and prevention of diabetes. All indices of GV and percentage of both TAR (180) and TAR (140) were higher in the prediabetes group than in the healthy group, but a significant difference was observed only in MAGE. Kishimoto et al. reported that the median CV, TAR (140) and TAR (180) were 18.3%, 10.4% and 0.6% in Japanese obese middle-aged men without diabetes [30]. Chakarova et al also found a significantly higher CV (20%) and significant increase of both SD and MAGE after adjustment for BMI, in the prediabetes group in comparison with the normal glucose tolerance group [31]. Our results were almost similar to those of these studies involving adults who were older and had higher BMIs than ours. Most interestingly, the underweight participants had the significantly higher values in SD and CV, compared to those with normal weight, in spite of lower mean glucose concentration. In Japan, it has been reported that young women tend to lose weight over the 25-year period [32]. According to Results of Year 2019 National Health and Nutrition Survey by Japanese Ministry of Health, Labor and Welfare [33], the percentage of people in their 20s with underweight has increased significantly in recent years, reaching the 20% level. Also, young underweight Japanese women had the higher prevalence of impaired glucose tolerance (IGT) than the normal weight women (13.3% vs 1.8%) and showed a lower insulinogenic index [34]. Together, these results suggest that the inability of β-cell function to compensate for decreased insulin sensitivity may contribute to the development of DM, especially in underweight women. In addition, the more muscle mass involved in glucose uptake, the better insulin resistance and the lower risk of prediabetes or overt diabetes [35]. Therefore, underweight women with lower average muscle mass may have severe insulin resistance in skeletal muscle. Thus, impaired insulin secretion and insulin resistance in skeletal muscle could be characteristic of underweight individuals with higher GV indices, resulting in postprandial hyperglycemia.

The mean caloric intake and daily activity in young women in Japan is very low and their daily steps count tends to be lower for these ten years [33]. Most participants (35 out of 40) including young women had the mean energy intakes lower than the estimated energy requirement for those with physical activity level I (low) in the “Dietary Reference Intakes for Japanese” by the Ministry of Health, Labor and Welfare [33]. No participants ate more than the recommended amount of carbohydrates per day (50–65% energy) [33]. On the other hand, as its recommendation regarding the amount of daily physical activity [36], it is considered ideal to secure "10,000 steps a day." The average step counts for this entire cohort was 9,264, similar to the underweight group (8,648), while the overweight group had a mean number of 6,394 steps, which was significantly lower. We therefore concluded that the higher GV in the underweight or overweight group was likely due to irregularity of eating habits or reduced daily physical activity. Regarding eating behaviors, the underweight eat more confections (73.4 g/d), but they were less aware of it (2.0 point). It was also found that the overweight tended to consume more sugar sweetened beverages (106.5g/d), but they didn’t pay much attention to it (1.5 point). In this study, we evaluated the factors associated with postprandial hyperglycemia in each of the three groups by BMI. Similarly, individual assessments are expected to identify factors that contribute to the individual’s postprandial hyperglycemia, and respective advice will help reduce glycemic excursions. Although lifestyle factors, including diet and physical activity, are often interrelated, interventions tend to focus on changing one health behavior rather than concurrently intervening on multiple behaviors. A meta-analysis of effects on glycemic control showed that lifestyle modifications based on physical or dietary intervention or both are associated with improvements in the 2-hour plasma glucose in IGT patients [37]. Much work is still needed to better understand how lifestyle factors may uniquely contribute to GV, and additional high-quality research on interventions designed to modify lifestyle behaviors is required to control blood glucose levels in healthy individuals.

Some limitations of this study deserve comments. Since the original research was exploratory, no power or sample size was calculated during the study design. The small proportion of participants limited comparisons of GV and life-related factors according to BMI classification. Sex and /or age-based analyzes were also not possible. However, this feasibility study confirmed that data collection from CGM, wearable devices, and questionnaire is sufficiently achievable. Secondly, this cohort of healthy individuals recruiting through internal company communication might limit generalizability. Thirdly, the overweight group in this cohort had an average BMI of 26.2 kg/m^2^, close to that of the normal weight group in other studies. Fourthly, both dietary questionnaires were surveyed at the end of the study, which may have introduced recall bias, albeit for short-term memory within 14 days. Finally, we could not estimate insulin secretion and insulin sensitivity based on the OGTT. Regardless of these limitations, our results outline clear tendencies and show the necessity of future long-term research focused on the association between lower BMI and GV. The high data acquisition rate in this study seems to warrant the feasibility of future large-scale studies.

## Conclusion

Significantly higher glycemic variability in SD and CV was observed for the underweight participants in comparison with the normal, which can be partly attributed to irregularity of eating habit (eating habit between meals) in free-living conditions. Future research is needed to determine which types of subjects with low BMI may have delayed insulin secretion and to identify the influence of low BMI on the development of IGT and/or T2DM. Concurrent CGM with diet and activity monitoring can yield health benefits to assess the impact of foods that individuals might consider healthy and to raise awareness of activity in daily living, i.e., enabling the practice of personalized medicine. These data also provide basic information for considering future treatments such as diet and exercise to predict or prevent the onset of diabetes.

## Supporting information

S1 File (XLSX)

S1 Table Summary of CGM metrics by BMI classification according to Asian-Pacific obesity guideline in healthy non-diabetes participants (n = 40). (DOCX)
