# Supplementary material for: Assessment of glycemic variability and lifestyle behaviors in healthy nondiabetic individuals according to the categories of body mass index
Source: PLoS One. 2023 Oct 4;18(10):e0291923. doi: 10.1371/journal.pone.0291923 (PMC10550127; doi:10.1371/journal.pone.0291923)
Supplement: S1 Table — (DOCX) [file pone.0291923.s002.docx]

| **S1 Table　 Summary of CGM metrics by BMI classification according to Asian-Pacific obesity guideline in healthy non-diabetes participants (n = 40)** | | | | | | | | |
| --- | --- | --- | --- | --- | --- | --- | --- | --- |
| BMI | Underweight  (< 18.5) | Normal weight  (18.5 ≦ < 23) | Overweight  (≥ 23) | | **P** | | | |
| **Characteristics of participants** | Gr1 (n = 5) | Gr2 (n = 27) | Gr3 (n = 8) | | Gr1 vs Gr2 | | Gr1 vs Gr3 | Gr2 vs Gr3 |
| Male (%) | 0 (0%) | 14 (52%) | 6 (75%) | | 0.043 | **0.016** | | 0.228 |
| Age, year | 35.4 ± 11.3 | 39.1 ± 11.8 | 47.4 ± 11.3 | 0.520 | | | 0.090 | 0.088 |
| BMI, kg/m^2^ | 17.8 ± 0.6 | 20..8 ± 1.4 | 25.0 ± 1.6 | **0.000** | | | **0.000** | **0.000** |
| FBS, mg/dL | 86.6±6.8 | 89.1±11.4 | 96.9±12.7 | 0.639 | | | 0.085 | 0.108 |
| HbA1c, % | 5.1 ± 0.3 | 5.2±0.4 | 5.4±0.3 | 0.359 | | | 0.060 | 0.333 |
| Prediabetes (%) | 0 (0%) | 10 (37%) | 4 (50%) | 0.131 | | | 0.057 | 0.398 |
| **Overall glucose distribution and variability** | | | | | | | | |
| Mean, mg/dL | 105.8 ± 5.2 | 108.9 ± 9.4 | 108.1 ± 9.3 | 0.482 | | | 0.588 | 0.824 |
| SD, mg/dL | 23.7 ± 3.5 | 19.8 ± 3.6 | 22.2 ± 5.7 | **0.034** | | | 0.607 | 0.292 |
| CV, % | 22.6 ± 4.6 | 18.2 ± 3.0 | 20.4 ± 4.3 | **0.009** | | | 0.398 | 0.103 |
| MAGE, mg/dL | 56.6 ± 7.4 | 49.4 ± 9.7 | 56.0 ±13.4 | 0.125 | | | 0.929 | 0.129 |
| **Percentage of glucose sensor values** | | | | | | | | |
| TAR (180), % | 1.2 ± 0.4 | 0.9 ± 1.3 | 1.8 ± 2.1 | 0.640 | | | 0.573 | 0.173 |
| TAR (180) > 0, % | 5 (100%) | 13 (48.1%) | 5 (62.5) | **0.043** | | | 0.196 | 0.380 |
| TIR(70-180), % | 95.0 ± 4.6 | 98.3 ± 1.3 | 97.4 ± 2.5 | 0.192 | | | 0.251 | 0.364 |
| TAR (140), % | 8.2 ± 1.1 | 8.7 ± 7.3 | 10.0 ± 7.6 | 0.889 | | | 0.529 | 0.656 |
| TIR(70-140), % | 87.6 ± 5.2 | 90.5 ± 7.1 | 89.3 ± 7.1 | 0.394 | | | 0.663 | 0.668 |

CGM, continuous glucose monitoring; SD, standard deviation; CV, coefficient variation; MAGE, mean amplitude of glycemic excursions; TAR, time above range; TIR, time in range; TBR, time below range. Prediabetes was defined by the HbA1c level of 5.7 to 6.4% and /or impaired fasting glucose level of 100 to 125 mg/dL.
